# Towards Rigorous Eye-Tracking Methodology in Interdisciplinary Fields: Insights from and Recommendations for Tourism Research

**DOI:** 10.3390/jemr19020031

**Published:** 2026-03-12

**Authors:** Wilson Cheong Hin Hong

**Affiliations:** 1 Faculty of Innovative Hospitality Management, Macao University of Tourism, Macao SAR 999078, China; wilsonhong@utm.edu.mo; 2 Faculty of Creative Tourism and Intelligent Technologies, Macao University of Tourism, Macao SAR 999078, China

**Keywords:** eye tracking, visual attention, tourism, destination marketing, best practices, methodology

## Abstract

Eye-tracking methodology represents a young but rapidly growing approach in tourism research, offering a direct window into the cognitive processes driving tourism stakeholders’ behaviour. However, a critical gap remains between the rapid adoption of this tool and the methodological rigour required to interpret its neurophysiological data. This critical review synthesizes 23 empirical studies (2020–2025) from the destination marketing and branding domain to diagnose eye-tracking’s state-of-the-art application. Adopting the SALSA framework (Search, Appraisal, Synthesis, Analysis) augmented by PRISMA 2020 guidelines, this study systematically searched Web of Science and Scopus databases. Studies were appraised using an eight-dimensional quality rubric, assessing from theoretical grounding to experimental design to statistical rigour. Findings revealed a “tool-first” exploratory phenomenon, where the majority of studies relied on basic fixation metrics to infer complex psychological states such as “interest”, when they could imply other cognitive states. Furthermore, most reviewed studies failed to control for stimulus-level confounds (e.g., luminance, AOI size) and utilized inappropriate data-handling procedures and methods, such as the absence of data cleaning and treating count and binary data as continuous data. These, coupled with transparency deficits, undermined the validity of their conclusions. Hence, a Checklist for Eye-Tracking Rigour (CETR) and a methodological decision tree were developed to guide researchers towards confirmatory and neurobiologically grounded research. Findings also provided a framework for managers/practitioners to more accurately interpret eye-tracking studies.

## 1. Introduction

The traditional silos of academia are rapidly changing landscapes as researchers increasingly embrace interdisciplinary fields and methodologies to unravel complex human behaviours. One of the most prominent beneficiaries of this shift is eye-tracking technology, which has transitioned from a specialized tool in cognitive psychology to a ubiquitous method utilized across health science, engineering, linguistics, and business, among others [1,2]. This proliferation is largely driven by a technological “explosion” that has rendered video-based eye trackers more affordable, non-intrusive, accurate, and accessible than ever before [1]. Certainly, compared to its long-term application in psychology [3], tourism has only started harnessing eye-tracking’s potential in the recent decade [4]. The ability for eye-tracking devices to record gaze location across time and task has empowered a diverse array of scholars to explore the “eye-mind link”, which is the theoretical assumption that what the eye fixates upon is a direct, albeit complex, reflection of immediate mental processing [5].

Within this interdisciplinary landscape, tourism and hospitality research has emerged as a fertile ground for eye-tracking applications. Tourism is, at its heart, largely an industry of the optic, from potential travellers’ online searching [6] to their actual travelling experience [7]. Because tourism products are largely intangible (e.g., a collection of experiences yet to be had), potential travellers must rely almost exclusively on visual surrogates such as photographs, brochures, and digital interfaces to form destination images and purchase intentions [8,9]. These have made the study of visual attention not merely a sub-discipline of marketing, but the very foundation of understanding tourist behaviour. Whether analyzing how people find ways [7] or how information density affects website navigation [6], eye-tracking provides an objective, neurophysiological window into unconscious reactions that traditional self-report measures often miss [8,10].

However, the rapid democratization of this technology has outpaced the development of shared methodological standards, especially for less-mature applications in interdisciplinary fields such as tourism [1,11]. Eye-tracking research is especially vulnerable to this trend due to the steep learning curve but limited time and knowledge for researchers to fully understand the technical specifications, mechanism and rationale, and cautions in interpreting results [12,13]. For example, using stimuli of multi-page textual guides and those for videos requires vastly different techniques [11]. In tourism, where research often involves complex, nonlinear, multimodal objects, these challenges can be pronounced [11]. Thus far, reviews on tourism eye-tracking research are largely descriptive, e.g., [14,15], while a comprehensive and critical guide to more rigorous eye-tracking methodology based on what are commonly missing is still absent.

This review paper aims to bridge the gap between technological capability and methodological rigour. By synthesizing interdisciplinary insights and reviewing tourism destination marketing/branding study as an illustrative case, a structured roadmap is provided for researchers. The following research questions are asked:

***RQ1***: How is eye-tracking currently used in destination marketing/branding research?

***RQ2***: What are the common methodological errors and pitfalls in current eye-tracking destination marketing/branding studies, and how can they be avoided?

We move beyond a mere review of the existing literature to offer practical, instructional guidelines on avoiding common pitfalls. The goal is to empower researchers to move past the highly attractive eye-tracking visualizations and toward more valid and replicable findings that can robustly inform both theory and practice.

### 1.1. Foundations of Attention and Eye Movement

The human eye is often likened to a camera, but its capacity for high-resolution capture is remarkably narrow. Fine detail and colour vision are almost exclusively the product of the *fovea centralis*, a small depression in the centre of the retina where colour-sensitive cones are most densely concentrated [1,16]. Despite the eye possessing a total visual field of approximately 140 degrees, the fovea covers a mere 1 to 2 degrees of visual angle, roughly the size of a thumbnail viewed at arm’s length distance [1]. Outside of this central “sweet spot”, acuity drops precipitously into the parafovea and periphery, which primarily detect low-frequency information and motion [17].

As the fovea is so small, the eye must constantly rove across the environment to assemble a coherent mental image. This movement is categorized into three primary states: fixations, saccades and regressions [18]. *Fixations* are periods of relative stillness (typically 180–330 milliseconds) during which the brain actually processes visual information [17]. Conversely, *saccades* are rapid, ballistic jumps between fixation points. It is a common misconception that we “see” while our eyes move; in reality, the brain suppresses visual input during a saccade, rendering us effectively blind for those 30 to 50 milliseconds [1,19]. *Regressions* are backward visits (to a previous element). High rates of regression often signal confusion, re-evaluation or increased cognitive load as the brain attempts to resolve ambiguity [17,18]. These three major visual behaviours could be measured in *duration* and *counts*, representing temporal and spatial properties, respectively. Pattern-based metrics, such as *scanpaths*, denote both (temporal) visual order and (spatial) whereabouts of processing [18]. Other non-temporal metrics include *pupillometry*, where an increase in pupil diameter is often associated with emotional arousal [18,20], and *blinking*, where decreased frequency often signifies high engagement, while increased frequency can indicate fatigue or inattention [2,12].

For tourism researchers, understanding the physiological properties is vital for the correct interpretation of results. For example, if a participant skips something in a travelling brochure [21], it is not simply that they ignored it; their brain may have literally never “seen” it because the jump occurred right over the target. Furthermore, a metric can be understood in several ways: longer fixations typically indicate deeper cognitive processing [18,22], which can reflect either higher interest or processing difficulty, and they can also be a result of mindless wandering [23]. Hence, it is important to consider, for instance, both spatial and temporal measurement methods. Longer fixations but lower fixation counts are likely to reflect heightened interest [3], while increased durations, counts and regressions likely reflect processing difficulties [18] and only increased fixation duration probably indicates wandering or viewing fatigue [23]. Further, eye movement patterns exhibit substantial individual differences based on factors such as reading ability, cultural background, expertise, and cognitive style [24]. These differences become particularly salient in tourism contexts where participants may have varied backgrounds (e.g., education, travel experience, cultural familiarity). Thus, it is essential for the research design to consider these variabilities and the analyses to control for these potential confounders.

### 1.2. Oculomotor Control vs. Neurobiology in Viewing Tourism Stimuli

As seen above, translating visual behaviour into cognitive constructs can be tricky. To resolve these interpretational ambiguities, researchers must look beyond the behavioural surface to the underlying neural mechanisms that drive these movements. Eye movements are the efferent output of distributed neural networks that regulate attention, selection and arousal. In the context of viewing complex destination stimuli, such as a cluttered website or a landscape photograph, fixations represent the maintenance of visual attention on an interest area. This process is governed largely by the dorsal frontoparietal attention network, specifically the Frontal Eye Fields (FEF) and the Intraparietal Sulcus (IPS) [25]. The IPS is thought to contain a “priority map” of the visual field, integrating bottom-up salience (e.g., the bright colours of a logo) with top-down goals (e.g., the search for price information) [26]. When tourism researchers interpret fixation duration as a proxy for “interest” [27], they are effectively measuring the engagement of these cortical areas in processing visual detail. Consequently, distinguishing between fixations driven by stimulus salience versus those driven by internal goals requires experimental designs that can disentangle these competing neural inputs. This means controlling the visual salience and providing a goal-directed task e.g., [6], are vital. Conversely, if a participant is asked to freely view a stimulus (e.g., a website) that contains unintended prominent objects (e.g., a red “pay now” button), researchers may not be able to isolate whether the heightened fixation is a neural reflex to the prominent object or a cognitive decision.

In terms of saccades, the decision of *where* and *when* to move the eye is mediated by the Superior Colliculus (SC), a midbrain structure that makes the neural decision upon receiving competing signals from the cortex: some urging the eye to move toward a new target (excitatory), others suppressing movement to maintain focus (inhibitory) [27]. The SC operates on a “winner-take-all” mechanism; that is, the eye moves only when neural activity for a specific target crosses a firing threshold. In tourism marketing, this reveals the trade-off between *exploration* (making large jumps to scan information for gist) and *exploitation* (making small jumps to scrutinize specific details). Apart from the spatial properties, the latency of saccades is not random; it reflects the time required for the neural activity in the SC to reach a threshold for movement initiation. Therefore, analyzing saccadic amplitudes and velocities offers insights into the efficiency of visual search and the captivating power of marketing elements, beyond what simple fixation counts can reveal.

Finally, while pupil diameter is often used in tourism research to measure “emotional response”, it is actually a complex physiological signal controlled by the Locus Coeruleus-Norepinephrine (LC-NE) system [28]. The LC-NE system regulates the brain’s overall state of arousal, modulating how sensitive neural networks are to incoming information. Crucially, the pupil responds to three distinct inputs: (1) luminance (constricting in brightness, dilating in darkness); (2) cognitive load (dilating during difficult mental tasks); and (3) emotional arousal (dilating during excitement or stress). This creates a significant risk of confounding variables. For example, a tourist’s pupil may dilate not because a destination image is “attractive” (emotional arousal), but simply because the website interface is confusing (cognitive load) or the image is darker than the previous one (luminance). Therefore, without rigorous luminance controls and baseline corrections, attributing pupil dilation solely to marketing effectiveness ignores the fundamental neurobiology of the LC-NE circuit.

Since different brain states can produce the same eye movement, researchers’ neurological knowledge can help inform correct methodological decisions: it necessitates the normalization of stimulus luminance to isolate emotional arousal in pupillometry, the use of specific task instructions to disentangle top-down attention from bottom-up capture, and the analysis of saccadic patterns to reveal search efficiency. Ultimately, grounding research in oculomotor and neurobiological understandings can facilitate metric selection, moving beyond arbitrary/mimetic choices to a hypothesis-driven process to ensure objective-method alignment and accurate interpretations.

## 2. Methods

This review integrated the methods of state-of-the-art review and critical review [29], combining comprehensive coverage of current eye-tracking literature under a strand (i.e., state-of-the-art review)—destination marketing/branding—with critical evaluation of methodological contributions (i.e., critical review). Unlike traditional systematic reviews that focus primarily on aggregating descriptive findings, e.g., [30], this review emphasizes conceptual and methodological innovation by critically assessing the quality and contribution of each reviewed study’s eye-tracking method, identifying gaps in current practice, and proposing a protocol for advancing eye-tracking rigour in tourism research. This methodological choice ensured recency, comprehensiveness and criticality without losing sight of systematicity, transparency and reproducibility. Specifically, Grant and Booth’s [29] SALSA (Search, Appraisal, Synthesis, and Analysis) framework, augmented by Page et al.’s [31] PRISMA 2020 to ensure reproducibility, is followed and adapted:

- Search: Comprehensive searching of current literature (2021–2025) to capture state-of-the-art practices;
- Appraisal: Critical evaluation based on methodological contribution and alignment with established best practices from psychology and cognitive science, rather than formal quality grading;
- Synthesis: Narrative synthesis with tabular accompaniment to characterize the current methodological landscape;
- Analysis: Identification of conceptual contributions, methodological gaps, and prioritized considerations for future investigation.

### 2.1. Search Strategy and Identification

Comprehensive searches across two databases, done independently by the author and a research assistant, were conducted to compile studies regarding destination marketing eye-tracking research. The databases used included Web of Science and Scopus, selected for their comprehensive coverage and rigorous indexing of top-tier journals in destination marketing and tourism management research. To ensure studies are representative of the state-of-the-art research, only journal articles were included.

Keywords were mainly extracted from existing destination marketing/branding studies. A dual-keyword strategy was employed to ensure comprehensive retrieval of relevant studies. Method-specific keywords included: “eye-tracking”, “eyetracking”, “visual attention”, “eye movement”, “visual pattern”, “gaze behavior”, “eye-tracking experiment”, and “neurophysiological”. Tourism-specific keywords included: “destination marketing”, “destination positioning”, “destination branding”, “neuromarketing”, “destination image”, and “decision making”. Boolean operators were adapted for each database to optimize search sensitivity while maintaining specificity. The search was conducted on 8 December 2025 covering the period from 1 January 2021 to the search date.

Web of Science (WoS): TS = (“eye-tracking” OR “eyetracking” OR “visual attention” OR “eye movement” OR “gaze behavior” OR “neurophysiological”) AND TS = (“destination marketing” OR “destination branding” OR “destination image” OR “tourism decision making”)
Scopus: TITLE-ABS-KEY((“eye-tracking” OR “eyetracking” OR “visual attention” OR “eye movement” OR “gaze behavior”) AND (“destination marketing” OR “destination branding” OR “destination image”))

**Figure 1:**
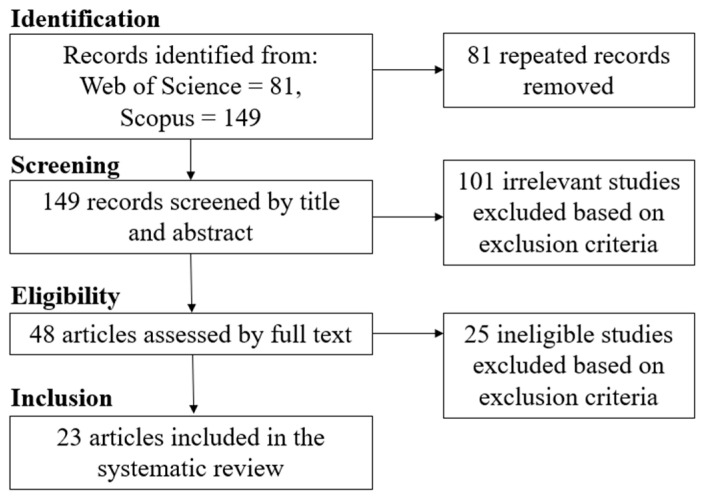
Flow diagram on article identification.

The initial search yielded 81 records in Web of Science and 149 records in Scopus. Then, duplicate records were eliminated manually, leaving 149 distinct records (see Figure 1). The deduplication log can be found in Supplementary Table S1. The titles and abstracts of these records were then examined by two independent reviewers to determine their relevance to the study’s objectives. Studies that did not meet basic inclusion criteria were excluded (see Table 1 for the inclusion and exclusion criteria), such as those unrelated to eye-tracking methodologies or tourism destinations. Inter-rater reliability was high, with disagreements occurring in only 2 cases (1.3%). These discrepancies were resolved through consensus-based discussion, resulting in the exclusion of both records. After this initial screening, 48 records remained that were considered potentially significant for detailed full-text assessment.

### 2.2. Final Selection and Appraisal

After applying the full-text assessment, 23 records were retained that directly addressed the current research questions regarding eye-tracking methodology in destination marketing research, with full agreement from the two reviewers.

Consistent with the critical review approach [29], studies were appraised based on their methodological contribution and alignment with established best practices, rather than through formal quality scoring, e.g., [32]. The appraisal process evaluated each study’s conceptual and methodological adoption across eight dimensions adapted from established eye-tracking methodology protocols [1,18,20], evaluating: (1) research design appropriateness, (2) apparatus selection and technical decisions, (3) theoretical foundation, (4) stimulus design and validation, (5) data collection procedures, (6) data quality and analysis methods, (7) statistical analysis appropriateness, and (8) reporting completeness. See Supplementary Table S2 for the detailed appraisal codebook.

Data extraction captured: study objectives, apparatus, eye-tracking measures, participants, theories adopted, stimulus type, validation methods, experiment design, procedure, data handling and data analysis. The initial use of NVivo was forfeited due the nested structure of the identified data (i.e., data required tabulation for further analyses) and the relatively objective methodological findings. Instead, manual coding was more efficient and thereby adopted.

## 3. Findings

Overall, the reviewed studies demonstrated a consistent upward trajectory in eye-tracking research applications within tourism destination marketing/branding, with annual publication counts increasing from 2 studies in 2021 to 7 studies in 2025. In fact, 2024–2025 represented over half of the identified studies (*n* = 12, 52%). Research predominantly originated from Asia (*n* = 15, 65%), particularly China (*n* = 11, 48%), followed by Europe (*n* = 7, 30%) and other regions (*n* = 1, 4%).

### 3.1. Eye-Tracking Technology and Measurement Selection

Studies employed diverse eye-tracking technologies, with desktop systems being most common (*n* = 10, 43%), followed by mobile/wearable trackers (*n* = 6, 26%), multimodal setups (*n* = 5, 22%), and online/webcam systems (*n* = 2, 9%). Popular brands included Tobii systems (*n* = 8, 35%), Gazepoint (*n* = 2, 9%), and various other platforms. Sampling rate was reported in 13 studies (56.5%).

In terms of metrics, fixation duration (*n* = 22, 96%) and fixation count (*n* = 18, 78%) were the most frequently measured metrics, while more sophisticated measures like pupil size (*n* = 5, 22%) and saccadic patterns (*n* = 2, 9%) were less commonly utilized. Heatmaps were generated in 15 studies (65%), while gaze trajectory analysis was conducted in 8 studies (35%). Technical specifications such as sampling rates varied considerably (See Table 2 for details on technical aspects).

### 3.2. Theoretical Foundations and Research Design

Twenty-two studies (96%) explicitly adopted theoretical frameworks, while one study (4%) lacked clear theoretical foundations. Among theoretically grounded studies, 15 studies (65%) employed social science theories, with Stimulus–Organism–Response (S-O-R) theory being most prevalent (*n* = 4, 17%). Six studies (26%) adopted psychological/cognitive theories that facilitate eye movement interpretations, including Processing Fluency Theory, Mental Simulation Theory, Information Processing Theory, Eye-Mind Hypothesis, and Multiple Resource Theory. One study (4%) utilized mixed theoretical frameworks combining both approaches.

Studies primarily focused on destination image perception (*n* = 8, 35%), visual attention patterns (*n* = 7, 30%), and marketing effectiveness (*n* = 6, 26%). Most studies employed between-subjects (*n* = 12, 52%) or within-subjects (*n* = 9, 39%) designs, with few utilizing mixed designs (*n* = 2, 9%). Sample sizes ranged from 23 to 548 participants (media *n* = 64), with most studies recruiting university students (*n* = 11, 48%) rather than actual tourists or diverse populations. Gender distribution was relatively balanced across studies, though age ranges were predominantly young adults (18–35 years). See Table 3 for research design details.

### 3.3. Integration with Other Methods

All studies (*n* = 23, 100%) employed multi-method approaches, demonstrating methodological triangulation. Six studies (26%) combined eye-tracking with cognitive/biometric measures including Electroencephalogram (EEG), Galvanic Skin Response (GSR), Functional Magnetic Resonance Imaging (fMRI), Electromyogram (EMG), and Electrodermal Activity (EDA). Fifteen studies (65%) integrated non-cognitive methods such as surveys, questionnaires, interviews, and member checking procedures to validate eye-tracking findings.

### 3.4. Stimulus Adoption and Validation

Stimuli adoption were often multimodal (*n* = 13, 57%), incorporating complex combinations such as text-plus-image, videos paired with textual mental simulation, and images embedded with natural sounds. Static images dominated stimulus types (*n* = 20, 87%), ranging from natural scenery photographs and AI-generated destination visuals to brand logos and social media screenshots. These were followed by texts (*n* = 10, 43%), which included linguistic signage and online reviews. Videos (*n* = 5, 22%) included 3D virtual scenes and intangible heritage performances. Additionally, audio and natural sounds (*n* = 4, 17%) were accompanied to examine visual attention and emotional changes.

Image-based studies typically used 18–60 stimuli, mostly repeatedly measured, while video studies employed 1–3 clips, mostly unrepeated. Nine studies (39%) reported stimulus validation procedures, such as expert review or pilot testing. Six studies (26%) controlled for visual properties like brightness, contrast, or colour composition (see Table 4).

### 3.5. Data Quality and Analysis Practices

Eleven studies (48%) reported specific data cleaning procedures, with common approaches including removing fixations below 80–200 ms thresholds, excluding outliers using statistical criteria, and discarding participants with poor calibration or insufficient attention. Calibration procedures were explicitly documented in only 5 studies (22%). Counterbalancing and randomization practices to reduce experimental effects were implemented in 14 studies (61%). Data transformation and normalization practices were reported in 11 studies (48%), including baseline corrections for pupil measurements, log transformations for normality, and normalized AOI data. Most studies employed basic statistical tests, while 7 studies (30%) utilized advanced techniques like mixed-effects modelling to control for unmanipulated confounders (e.g., participants’ individual visual differences).

## 4. Discussion

The integration of eye-tracking technology into destination marketing research reflects a significant shift toward understanding the unconscious cognitive processes that drive tourist behaviour [11]. Addressing RQ1 regarding current usage trends, the findings reveal a stable increase in adoption but a concerning reliance on basic fixation metrics and macro-level theories to explore rather than to confirm, often without accounting for the underlying cognitive processes to extract meaning behind the viewing patterns. For example, reviewed studies made minimal effort in distinguishing bottom-up salience from top-down goals that drive metric results. Specific to RQ2 regarding common methodological pitfalls, four prevalent systemic failures were identified: (1) theoretical and metric misalignment (65%), (2) lack of experimental control (74%), (3) statistical inadequacy (70%), and (4) transparency/reporting deficits (57%). These systematic pitfalls suggest that while the adoption of the technology is increasing, the sophistication of its application lags behind established standards in cognitive neuroscience, which may compromise the validity and reliability of findings. Below is a detailed breakdown of these areas of observation.

### 4.1. Theoretical Grounding and Hypothesis Testing

A fundamental recommendation for eye-tracking research is that it should move beyond descriptive observation toward theory-driven, confirmatory designs grounded in cognitive frameworks such as the Eye-Mind Hypothesis or Attention Resource Theory [2,55]. While 96% (22/23) of reviewed studies adopted theoretical frameworks, the majority (65%, 15/23) only adopted social science theories without referring to psychological/cognitive frameworks that facilitate eye movement interpretations. For instance, while Deng et al. [33] utilized the S-O-R framework to explain how environmental aesthetics evoke emotional responses, this macro-level theory provides limited guidance for interpreting millisecond-level fixation patterns or pupil dilations. In contrast, studies like Zhao et al. [39], which drew on Multiple Resource Theory to investigate modality interference, demonstrate superior theoretical alignment by connecting cognitive load theory directly to measurable eye movement parameters.

Further, the dominance of exploratory designs lacking a priori manipulation of independent variables undermines the field’s capacity to demonstrate true causality [2,11]. This theoretical misalignment becomes particularly problematic when researchers attempt to infer complex cognitive processes from basic fixation metrics without grounding in cognitive frameworks that explicitly link attention and lexical processing to eye movement control [18,55]. As stated, the same eye movement can entail different meanings: long fixation duration can be understood as heighted interest [3], or processing difficulties [18] or just mindless wandering [23]. Hence, apart from social science theories, tourism researchers are recommended to explain results in light of micro-level processing theories.

### 4.2. Metric Selection and Cognitive Process Alignment

The overwhelming reliance on the two metrics of fixation duration (96%, 22/23) and fixation count (78%, 18/23) reveals a concerning oversimplification of the rich data available through eye-tracking technology. Fixations are connected to activities in the dorsal frontoparietal network (specifically the FEF and IPS), which governs the maintenance of attention [26]. However, they alone cannot distinguish whether the priority in the IPS is being driven by bottom-up stimulus salience or top-down cognitive goals [27]. From a neural perspective, this over-reliance is problematic because high fixation density can signal two opposing cognitive states: deep engagement (positive interest) or high processing difficulty (cognitive load). Hence, while these basic metrics are relatively accessible and seemingly straight-forward, they often fail to capture the highly refined cognitive processes central to tourism decision-making [11]. The underutilization of more sophisticated measures like regressions (22%, 5/23) and saccadic patterns (9%, 2/23) represents a missed opportunity to investigate the Superior Colliculus (SC) mechanisms that mediate the trade-off between exploration and exploitation [28]. Thus, studies are unlikely to capture the cognitive load and information processing strategies that are central to viewing behaviour [18].

More critically, many studies demonstrate a fundamental misalignment between stated research objectives and chosen measures. Studies investigating “emotional responses” or “cognitive engagement” frequently rely on fixation duration, yet only 22% (5/23) incorporated pupillometry. This is a significant gap because the pupil provides a direct window into the Locus Coeruleus-Norepinephrine (LC-NE) system, which modulates arousal and sensitivity to information [28]. Only incorporating pupillometry or physiological measures could provide direct evidence of arousal or emotional changes [1]. This metric–objective mismatch undermines the validity of conclusions drawn about complex psychological processes. Further, not all studies justified for the adopted metrics, and the majority (65%, 15/23) that did only provided superficial or less-than-accurate justifications. For example, Shi et al. [35] and Pike et al. [37] explained fixation counts as an indicator of “importance”, while Guo et al. [34] and Zhao et al. [39] justified that fixation duration manifested “interest”. Overall, metrics appear to be either adopted by (unfounded) conventions or a posteriori selections, without showing adequate understanding of what each adopted metric entails. The field would benefit from following standardized metric selection protocols that justify measures a priori to prevent p-hacking and ensure theoretical coherence [2,18].

### 4.3. Stimulus Precision and Modality-Specific Controls

It is crucial to emphasize that different stimulus modalities require distinct technical approaches and controls. Text-based stimuli demand high spatial precision, ideally using monospaced fonts and double-spacing to ensure fixations map accurately to specific words [18,56]. While Chang et al. [48] investigated the impact of calligraphic versus printed fonts on visual attention, demonstrating awareness of these requirements, many studies utilize social media screenshots or website interfaces where font styles are irregular and difficult to control, such as the online reviews used by Bigne et al. [44]. Further, the majority (74%, 17/23) of studies did not use other means (e.g., statistical methods) to mitigate these random differences.

For static images, researchers must account for visual salience (size, colour, and luminance), as “eye-catching” elements can attract attention regardless of the task’s relevance [1,11]. The finding that only 26% (6/23) of studies controlled for visual properties represents a critical methodological gap. Ye et al. [47] addressed this by normalizing pupil diameter measurements against a baseline to account for luminance, but this level of technical control is often absent in studies using “organic” images sourced from travel platforms like Ctrip or Instagram, e.g., [45,51]. Better still, the Areas of Interests (AOIs) sizes should be standardized, e.g., [36]. The general lack of stimulus control introduces confounding variables that can “swamp” the subtle cognitive effects researchers aim to measure, particularly when investigating emotional responses through pupillometry where luminance effects can overwhelm small emotional dilations [1]. Overall, the added ecological validity using unmanipulated stimuli should not be at the expense of fundamental rigour.

### 4.4. Ecological Validity vs. Technical Precision Trade-Offs

Along this line of discussion, the tension between ecological validity and technical precision represents one of the most challenging methodological decisions in tourism eye-tracking research. Best practices suggest that desktop trackers with chin rests are essential for tasks requiring millisecond accuracy, such as reading [1]. However, tourism research often prioritizes familiar settings or naturalistic environments to ensure authentic behaviour [11]. A total of 43% (10/23) of the reviewed studies used desktop trackers and with sufficient reporting of technical setups, e.g., [41,52], which suggests precision of tracking is quite often not prioritized by tourism researchers. Such studies will require reasonable research objectives, with accounts of limitations and clear theoretical justification for the methods to be valid.

**Figure 2:**
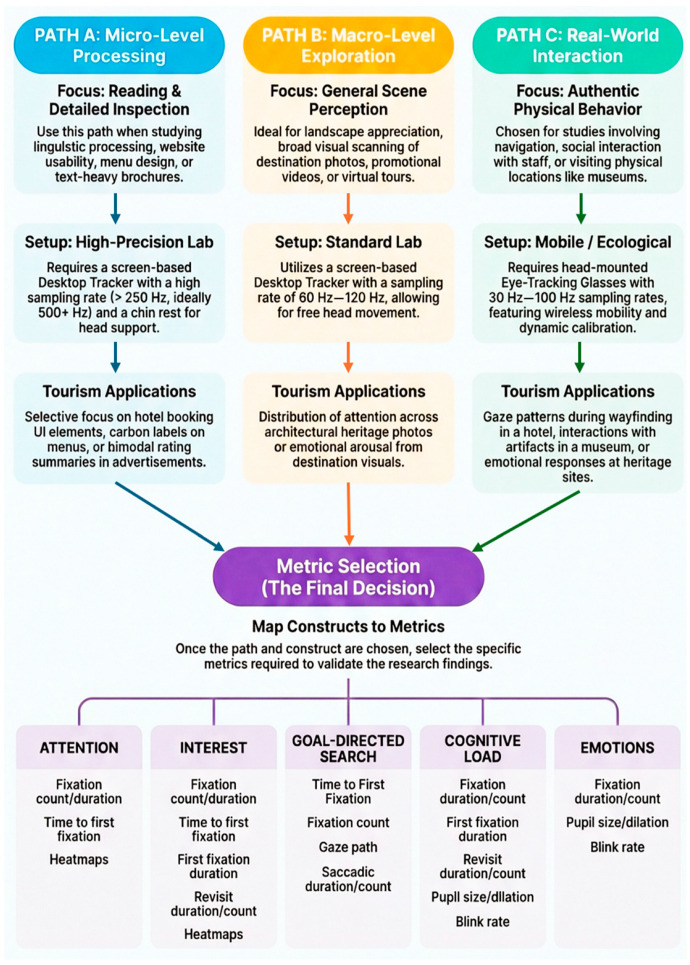
Methodological decision tree.

Studies by Li et al. [40] exemplify how valid research can be conducted when the objective is not micro-level linguistic processing, but rather the broader capture of tourist responses to complex environmental stimuli. They opted for the Pupil Core eye-tracker, a mobile glasses-mounted device, to better reflect authentic visual experience. Rather than purely exploratory attempt, Li et al. utilized Attention Restoration Theory to examine stress in nature. A “balanced” approach was taken by conducting the experiment in fixed lab settings, but with images presented on a large screen to more accurately simulate a tourist’s field of view in a geological park. Data were properly cleaned. Details accounts are given as to methodological decisions, and limitations regarding the discrepancy between real settings and the lab environment are duly provided. Nevertheless, sampling rates and other technical specifications were insufficiently justified or reported in 57% (13/23) of the reviewed studies, which may suggest many researchers were unaware of how these technical decisions impact data quality and interpretability. To facilitate researchers’ decisions, a visualized methodological decision tree is presented for researchers’ reference (Figure 2).

### 4.5. Data Quality and Statistical Analysis Sophistication

The finding that less than half (48%, 11/23) of the studies reported specific data cleaning procedures represents a fundamental threat to research reproducibility and validity. The eye-tracking technique is prone to artefacts [18]. Rigorous data cleaning procedures, including explicit description and justification of outlier removal and reporting of total data loss percentages, are prerequisites for credible eye-tracking research [1,56]. Only 22% (5/23) of studies reported calibration, e.g., [32,39,42], which is particularly concerning, as accurate data collection depends entirely on high-quality calibration at the start of every session [1].

The statistical analysis approaches reveal another critical gap. While 30% (7/23) of studies utilized advanced techniques like mixed-effects modelling, the majority (70%, 16/23) relied on basic statistical tests that fail to account for the nested data structures and individual variability inherent in eye-tracking research [2,18]. Mixed-effects modelling is essential for handling variability in both individuals and stimuli, yet many studies treat eye-tracking data similar to survey data, which increases the likelihood of Type I errors (i.e., false p-significance) [2,56]. The majority of eye-tracking experiments reviewed were comparison-based and involved repeated measurement, which they rightly did, but the relatively smaller sample sizes would mean the analyses were more susceptible to individual and stimulus differences. Hence, the use of mixed-effects modelling to control for these random factors [2,57], typically with R or Python-based packages, is highly recommended. These packages can also more robustly handle “count” data [e.g., fixation count, revisit count], which theoretically should not be analyzed like “duration” data, as was done in all the reviewed studies, since they are of discrete data type [58]. Hence, Poisson regression or negative binomial regression, instead of linear regressions, are more appropriate models of analysis. A quick guide is provided in Table 5 on the data type and recommended distributions.

### 4.6. Data Triangulation and the Purpose of Attention

Because gaze fixations do not always imply high-level cognitive processing, eye-tracking should be complemented with other methods. In psychological investigations, eye-tracking combined with other biometric methods such as fMRI is common [3]. A few (26%, 6/23) of the current tourism studies that focused on nuanced processing mechanisms and differences, e.g., [38,50] triangulated eye-tracking with fMRI or EEG. If resources allowed, this method to link visual attention to neural activations can most safely inform cognitive changes or differences. Nevertheless, this high-cost equipment is often less accessible, and therefore, retrospective surveys or interviews to capture the “purpose” of attention [11,12] can be conducted instead. The finding that 100% (23/23) of studies employed some of these multi-method approaches is encouraging. However, the timing of such self-reported method administration is critical. Few studies have reported short eye-tracking-task with immediate self-reports, e.g., [36]. If the eye-tracking experiment is lengthy, researchers can break it into smaller tasks followed by the survey or interview. Alternatively, researchers have recommended adopting the think-aloud protocol [2,56] during the experiment, with participants providing real-time “commentary” as they view the stimuli. These can increase confidence in interpreting the eye-tracking results.

### 4.7. Reporting Standards and Reproducibility Issues

Complete methodological reporting, including tracker model, sampling rate, and calibration error, is a prerequisite for replicability [1,18]. While 96% (22/23) of studies reported equipment specifications, the inconsistent documentation of critical technical parameters and procedures makes destination marketing/branding studies using eye-tracking hard to reproduce. This reporting gap is particularly concerning given the rapid evolution of eye-tracking technology and the proliferation of low-cost, web-based systems [46]. While most reviewed research provided basic hardware descriptions, the vital information of experiment setup, thresholds (e.g., calibration, acceleration, artefacts), data inspection and cleaning procedure was often unreported [1,2]. For instance, sampling rates were explicitly reported in only 43% (10/23) of studies. Studies that followed good practices include Shi et al. [35], which noted the exclusion of fixations below 200 ms, and Chang et al. [48], which reported a bidirectional offset of less than 0.5° to ensure spatial accuracy. Few studies detailed apparatus setup, such as sampling rate, e.g., [41], viewing distance and monitor resolution, e.g., [38,52].

On the other hand, caution should be taken in certain reporting. Heatmaps are the third most commonly reported results (65%, 15/23) in the reviewed studies, but are often misidentified as an analysis method, when they primarily serve visualization or exemplification purposes [20]. While these colourful maps are “eye candies” for readers and editors alike [2], their interpretation can be highly subjective and at times challenging [11]. Hence, they cannot replace inferential statistical analysis of quantitative metrics and should not be over-reported and interpreted. As some good examples, Li et al. [45] and Xu et al. [42] utilize heatmaps to (qualitatively) identify areas viewed most intensively but rely on ANOVA and correlation models of fixation counts and durations to provide evidence of tourist preferences. Better still, researchers are recommended to explicitly state the heatmap figures are for illustrative purposes so that readers will not be misled into believing that they are the visual patterns of all participants.

The field of tourism calls for standardized reporting protocols, similar to those established in psycholinguistics and cognitive psychology [18,56], to advance the scientific credibility of eye-tracking research.

## 5. Conclusions

The eye-tracking methodology is sophisticated and takes time to familiarize [13]. The mentality to adopt eye-tracking in an opportunistic manner, using it as a one-off “fancy” instrument without readiness to invest sufficient time to learn and understand it, should be avoided. Based on this state-of-the-art critical review of eye-tracking methodology in tourism destination marketing and branding research, nine critical recommendations emerge to enhance scientific rigour and validity:

1. Adopt cognitive-level theoretical frameworks: Move beyond macro-level social science theories toward psychological frameworks that directly inform eye movement interpretation.
2. Implement theory-driven confirmatory designs: Prioritize a priori manipulation of independent variables and hypothesis-testing over purely exploratory investigations.
3. Justify metric selection a priori: Align eye-tracking measures with research objectives; incorporate pupillometry for emotional responses, regressions for processing effort, saccadic patterns for cognitive load, rather than relying solely on basic fixation metrics.
4. Implement modality-specific controls: Use monospaced fonts and high sampling rates (>250 Hz) for text, control visual salience (size, colour, luminance) for images through pilot testing and statistical controls, define time-dependent AOIs (manual keyframe interpolation or automated tracking) and consider the effect of audio.
5. Balance ecological validity with technical precision: Choose desktop trackers for high-precision tasks, mobile systems for naturalistic studies, with justification for equipment selection.
6. Adopt advanced statistical analyses: Use mixed-effects modelling as standard practice to account for individual and stimulus variability inherent in eye-tracking data.
7. Implement rigorous data quality protocols: Report data cleaning procedures, calibration standards, and data loss percentages. Establish clear exclusion thresholds and inspect raw data systematically.
8. Enhance methodological transparency: Report complete technical specifications (tracker model, sampling rate, viewing distance, calibration thresholds, processing algorithms) following standardized protocols.
9. Implement systematic data triangulation: Complement eye-tracking with neuroimaging (fMRI, EEG), retrospective interviews, think-aloud protocols, or immediate post-task surveys.

To facilitate methodological rigour, a fillable Checklist for Eye-Tracking Rigour (CETR) is provided in the Appendix A Table A1. Tourism researchers are recommended to tick and provide the location of all items and submit this form alongside their manuscript.

### 5.1. Practical Implications

While this review focuses on methodological rigour, the findings have direct implications for destination management organizations (DMOs) and marketing practitioners who increasingly rely on eye-tracking (research or marketing reports) to evaluate promotional materials.

First, the inherent ambiguity between salience and goal (Section 4.3) suggests that managers should be cautious when interpreting heatmaps of unmanipulated stimuli. A “hotspot” on a brightly coloured Instagram ad feature may simply reflect a bottom-up response to a visually salient element, rather than genuine top-down interest in the destination, much less reflecting the ad’s effectiveness. By adopting the rigorous controls suggested in this review, practitioners can better distinguish between “eye-catching” clutter and “mind-engaging” content, leading to more efficient allocation of advertising budgets.

Second, the emphasis on appropriate metric-adoption, triangulation and rigorous data handling (Section 4.2, Section 4.5 and Section 4.6) provides a roadmap for measuring emotional “arousal” more accurately. Because eye-tracking data are inherently noisy and subject to significant individual differences, such as varying baseline pupil sizes or idiosyncratic viewing patterns, proper experimental designs and data handling allow for a deeper, more reliable understanding of the valence of tourists’ reaction. This precision is vital for DMOs when selecting campaign imagery that resonates across diverse audiences.

### 5.2. Limitations

This review has several limitations that should be acknowledged. First, the temporal scope was restricted to the period of 2021–2025 to capture the most recent methodological advancements. While this focus allowed for a deep critique of state-of-the-art practices, it precludes a historical analysis of the field’s evolution over the past decade. Future bibliometric reviews could extend this timeframe to examine the long-term trajectory of eye-tracking applications in tourism. Second, the search was limited to Web of Science and Scopus, which, while comprehensive for high-impact marketing journals, may have omitted relevant technical papers and non-English articles indexed in other databases. Relatedly, the relatively small number of eligible studies (*n* = 23) may not fully represent the breadth of approaches being employed in the field. However, a conscious decision was made to investigate the author’s specialized area (i.e., destination marketing/branding) and to use well-regarded databases and more recent studies to represent state-of-the-art tourism eye-tracking methodology. Future bibliometric/scope reviews could examine the decadal evolution of this and other strands of tourism research. Knowingly, this review’s emphasis on established best practices from psychological and psycholinguistics disciplines may not fully account for the unique contexts, methodological challenges, and opportunities specific to tourism research.

## Figures and Tables

**Table 1:** Article inclusion and exclusion criteria.

Inclusion Criteria	Exclusion Criteria
1. Empirical eye-tracking studies with clearly reported methodology 2. Focus on destination marketing and related customer decision-making phenomena 3. Investigation of visual attention, gaze behaviour, or processing by means of eye-tracking 4. Peer-reviewed journal articles 5. English-language publications 6. Studies published between 2021 and 2025	1. Studies that were not empirical (theoretical articles, reviews, or commentaries) 2. Studies published as preprints, conference proceedings, extended abstracts, book chapters or dissertations 3. Studies that did not focus on eye-tracking methodologies or applications in destination marketing 4. Studies mentioning eye-tracking but without doing relevant experiments 5. Research examining atypical (e.g., pathological) eye movements

**Table 2:** Technical specifications and eye-tracking metrics adopted.

			Fixations				Regressions		Saccades		Physiology		Patterns	
Author(s)	Year	Apparatus (Manufacturing Info.), Reported Sampling Rate	Fixations Duration	Fixation Count	First Fixation Duration	Time to First Fixation	Revisit Duration	Revisit Count	Saccadic Duration	Saccadic Count	Pupillometry	Blink Rate	Heatmap	Scanpath
Deng et al. [33]	2021	SMI Hi-Speed System (SensoMotoric Instruments, Teltow, Germany), 500 Hz	X	X	X	X			X		X			X
Guo et al. [34]	2021	SMI Mobile (SensoMotoric Instruments, Teltow, Germany), NA	X	X									X	X
Shi et al. [35]	2022	Dikablis Head-Mounted (Ergoneers GmbH, Manching, Germany), 50 Hz	X	X										
Hong et al. [36]	2022	Gazepoint GP3 HD (Gazepoint Research Inc., Vancouver, BC, Canada), 150 Hz	X	X		X		X						
Pike et al. [37]	2022	Tobii Pro TX300 (Tobii AB, Stockholm, Sweden), 120 Hz	X	X										
Savelli et al. [38]	2022	Eye Tribe (The Eye Tribe, Copenhagen, Denmark), 60 Hz	X			X							X	
Zhao et al. [39]	2022	Tobii Pro X2-60 (Tobii AB, Stockholm, Sweden), 60 Hz	X	X									X	
Li et al. [40]	2023	Pupil Core (Pupil Labs GmbH, Berlin, Germany), 250 Hz	X	X									X	
Xie et al. [41]	2023	EyeLink 1000 Desktop (SR Research Ltd., Ottawa, ON, Canada), 1000 Hz	X		X						X			X
Xu et al. [42]	2023	Credamo HBO Platform (Credamo, Beijing, China), NA	X	X	X	X							X	X
Zhu et al. [43]	2023	VR Helmet & Micro Tracker (Unspecified Integrated System), NA	X	X										
Bigne et al. [44]	2024	Unspecified eye-tracker, NA	X	X		X		X					X	
Calderón-Fajardo et al. [21]	2024	Unspecified Tobii tracker (Tobii AB, Stockholm, Sweden), NA	X	X	X	X							X	X
Li et al. [45]	2024	Tobii Pro X2-60 (Tobii AB, Stockholm, Sweden), 60 Hz	X	X									X	
Wasaya et al. [46]	2024	RealEye.io Webcam (RealEye, London, UK), NA	X										X	
Ye et al. [47]	2024	Tobii Pro Fusion (Tobii AB, Stockholm, Sweden), NA	X	X						X	X	X		
Chang et al. [48]	2025	Tobii Pro Spectrum (Tobii AB, Stockholm, Sweden), 300 Hz	X	X	X	X							X	X
Chang et al. [49]	2025	Tobii Pro Spectrum (Tobii AB, Stockholm, Sweden), 300 Hz	X	X	X	X	X	X					X	
García-Carrión et al. [50]	2025	EyeLink 1000 Plus Desktop (SR Research Ltd., Ottawa, ON, Canada), 500 Hz	X	X					X		X	X		
Jin et al. [51]	2025	Pupil Core (Pupil Labs GmbH, Berlin, Germany), NA	X	X			X						X	
Jóźwiak [52]	2025	Gazepoint GP3 HD (Gazepoint Research Inc., Vancouver, BC, Canada), 150 Hz	X	X		X		X					X	X
Li et al. [53]	2025	Tobii Pro Glasses (Tobii AB, Stockholm, Sweden), NA											X	
Sushchenko et al. [54]	2025	Realeye.io/Gazerecorder (RealEye, London, UK), NA	X			X				X	X		X	X

NA = Not available.

**Table 3:** Study designs.

Author(s)	Year	Study Objectives	Participants	Theories Adopted	Other Methods
Deng et al. [33]	2021	To investigate how visual aesthetics in tourism photography influence destination choice intention.	64 Chinese students (28 female), ages 20–30.	Stimulus–Organism–Response (S-O-R).	Visual appeal/emotion/impression/intention survey
Guo et al. [34]	2021	To measure the impact of artificial elements on the visual perception and value assessment of mountain landscapes and destination image.	96 mostly Chinese students (47 female), ages unspecified.	Environmental Cognitive Psychology (Top-down/Bottom-up).	Value assessment scale survey
Shi et al. [35]	2022	To explore how tourists recognize tourism rituals through destination images to stimulate travel motivation.	60 Chinese adults (30 female), ages under 24 to over 55.	Push–Pull Theory; Generation Theory.	Push/Pull motive survey
Hong et al. [36]	2022	To examine how exotic landmarks affect the visual attention and visiting intention of young travellers.	40 Chinese and English-speaking travellers (20 female), ages 18–25.	Novelty Seeking & Push–Pull Theory.	Post-experiment intention survey
Pike et al. [37]	2022	To determine the relative importance of stopover destination attributes and destination choices.	110 Australian residents (73 female), ages 18–35+.	Determinant Attribute Theory.	Stated importance, Discrete Choice Experiment
Savelli et al. [38]	2022	To identify which communication signals are most effective at capturing attention for food tourism destination promotion.	40 Italian travellers (24 female), ages 18–65.	Signalling Theory.	EEG (Engagement, Cognitive load)
Zhao et al. [39]	2022	To examine the effect of spatial displacement and modality on tourist attention and travel intention.	120 Chinese students (60 female), ages 18–26.	Multiple Resource Theory (Modality Interference).	Travel intention Likert-type scale survey
Li et al. [40]	2023	To investigate visual attention and stress under varying factors in nature-based destinations.	50 Chinese tourists (27 female), ages 19–57.	ART; Transactional Theory of Stress.	Stress intensity scale survey
Xie et al. [41]	2023	To examine how guided mental simulation of an upcoming trip influenced by marketing information affects subsequent on-site experience evaluations.	121 Chinese students (71 female), average age 21.	Mental Simulation Theory.	Engagement and experience scale survey
Xu et al. [42]	2023	To explore the impact of virtual tourism content and form on visual appeal and travel intention.	210 Chinese adults (106 female), ages 18–35+.	S-O-R; Cognition-Affection-Conation (C-A-C).	Scenario authenticity survey
Zhu et al. [43]	2023	To reveal the influence of virtual social elements in VR environments on social perceptions and visit intention.	254 Chinese students/teachers (118 female), average age 23.38.	S-O-R; Para-social Interaction.	S–O-R scale survey, PLS-SEM analysis
Bigne et al. [44]	2024	To assess how online review types and brand familiarity impact destination informativeness and persuasiveness to travel.	548 Spanish residents (312 female), ages 18–54+.	Persuasion Schema; ATF; Commitment-Trust.	EEG (Frontal alpha asymmetry)
Calderón-Fajardo et al. [21]	2024	To analyze how destination logos and AI-generated visuals influence attention and emotional responses towards destination branding.	40 mostly Spanish students (20 female), average age 23.25.	Brand Personality.	Galvanic Skin Response (GSR)
Li et al. [45]	2024	To identify the categories and specific elements of Intangible Cultural Heritage that attract the most visitor attention.	45 Chinese students (23 female), average age 22.	Information Processing Theory.	Retrospective oral reports
Wasaya et al. [46]	2024	To examine how norms, personality, and preference affect a tourist’s place attachment to heritage destinations.	453 mostly Australian travellers (137 female), ages 18–56+.	Social Congruity; Normative Conduct.	Personality scale, facial emotion analysis
Ye et al. [47]	2024	To investigate the relationship between eye-tracking and emotional experiences regarding traditional village destinations.	53 Chinese students (40 female), average age 22.	Eye-Mind Hypothesis.	mDES emotion scale survey
Chang et al. [48]	2025a	To examine the role of fonts and practical subjects in how linguistic landscapes impact travellers’ visual attention.	165 Chinese tourists (107 female), ages 18–55+.	Processing Fluency; Selective Attention.	Perception scale survey
Chang et al. [49]	2025b	To reveal the mechanism from visual attention to emotional experience toward destination linguistic landscapes.	165 Chinese tourists (107 female), ages 18–55+.	S-O-R; Emotional Evaluation.	EDA, EMG, and emotional scales survey
García-Carrión et al. [50]	2025	To examine the effect of message congruence on cognitive effort and behavioural responses across positioning strategies.	49 Spanish Facebook users (30 female), ages 24–40.	Customer-integrated marketing communications (CIMC) model; Cognitive Dissonance.	fMRI (Neural activity/BOLD signals)
Jin et al. [51]	2025	To explore attention patterns and effectiveness of destination marketing pictures based on sources and attribute compositions.	56 Chinese students/staff (39 female), adult population.	Dual-Process Theory; AIDA Model.	Perceived advertisement effectiveness survey
Jóźwiak [52]	2025	To examine visual attention to eco-oriented elements in advertising and their influence on choices.	23 Polish-speaking students, ages 18–22.	Marketing Communication; Visual Attention.	Post-exposure survey
Li et al. [53]	2025	To investigate if modernizing traditional Intangible Cultural Heritage can attract and build destination loyalty among young festival-goers.	320 Chinese festival attendees (232 female), ages 18–34.	Cognitive Appraisal Theory (CAT).	EDA measures (emotional peaks) and interviews
Sushchenko et al. [54]	2025	To identify the visual identity elements that most interest potential tourists for use in advertising campaigns.	Unspecified small Ukrainian student/staff sample, ages unspecified.	Nil	Post-experiment ease/attractiveness/accessibility survey

**Table 4:** Experiment details and data.

Author(s)	Year	Stimulus Type & Validation	Experiment Design & Procedure	Data Handling	Cognition-Related Significant Effects
Deng et al. [33]	2021	48 images (natural vs. built); colour/brightness processed; piloted for “professionalism”.	2 × 2 mixed design; 10 s display per image; random assignment/presentation.	Normal distribution was verified using skewness and kurtosis checks.	Fixation duration (+), Fixation Count (+), Pupil size (+), Saccadic duration (+)
Guo et al. [34]	2021	9 official mountain photos (long/medium shots).	2 × 3 within-subject; participants-controlled viewing time; random order.	Outliers/zeros excluded; log transformation for normality.	Fixation count (+), Fixation duration (+)
Shi et al. [35]	2022	48 New Zealand photos; Delphi-validated by 5 experts for “ritual modes”.	48 images; 10 s display; stratified random sampling.	Fixations < 200 ms excluded; last 15 images excluded for head stability; normal distribution verified.	Fixation count (+), Fixation duration (+)
Hong et al. [36]	2022	22 city pictures (exotic vs. Chinese); image size, colour composition and brightness normalized.	2 × 11 factorial design; images shown in pairs; calibration; random city sequence.	Normalized AOIs; mixed ANOVA for non-parametric analyses and controlling of confounders.	Fixation duration (+), Fixation count (+), Revisits (+)
Pike et al. [37]	2022	8-attribute stopover packages (text-based); validated by previous interview/survey.	Discrete choice experiment; 8 choice sets; vertical attribute order randomized.	Mixed logit models were estimated using simulated likelihoods to handle panel data.	Fixation counts (+), Fixation duration (+)
Savelli et al. [38]	2022	Graphic illustrations; focus group and Implicit Priming Test (IPT) validated.	2-step test: 10 s single image exposure, then 30 s simultaneous comparison.	Responses that were too quick (<250 ms) or too slow (>500 ms) were discarded via an internal algorithm. Fixations shorter than 80 ms were omitted as invalid.	Fixation duration (+), Time to first fixation (−), EEG Cognitive load (+), Engagement (+)
Zhao et al. [39]	2022	18 experimental + 6 interference pictures (Google travel).	4 × 3 between-subjects; 7 s presentation per picture; randomized order.	Calibration values were required to meet specific screen standards before data collection. Comparisons across different stimulus distributions utilized standardized scores.	Fixation duration (+), Fixation count (+)
Li et al. [40]	2023	Real-time observation photos (6 densities/PPV).	6 × 2 × 2 within-subjects; 10 s display; integrated natural sounds.	100 ms fixation threshold for data cleaning; visual attention calculated as a ratio of targeted to total viewing time to exclude saccades.	Fixation duration & count (crowded→people: +, landscape: −; natural sound→landscape: +, stress, −)
Xie et al. [41]	2023	1st-person videos (Peter Pan Flight vs. Museum).	2 × 2 between-subjects; 4-step data collection including baseline pupil check.	Baseline correction to pupil size to account for oscillations caused by fatigue or anxiety; 90–2000 ms fixation threshold; Multivariate ANOVA.	Fixation duration (+), Fixation count (+)
Xu et al. [42]	2023	1st-person videos and pictures (real vs. modelled virtual).	2 × 2 between-groups; pictures (4 s/ea) vs. video (29 s); random allocation.	Data cleaned by automatically excluding respondents who failed screening or calibration standards; Fixation time checked for consistency across different stimulus forms. Nonparametric ANOVA was utilized.	Fixation duration (+), Fixation count (+)
Zhu et al. [43]	2023	3 versions of VR Plaza Italia (modelled in Vizard).	Single-factor between-subject; participants toured freely; random assignment.	Single-factor ANOVA tests confirmed no significant demographic differences between experimental groups.	Fixation duration (+), Fixation count (+)
Bigne et al. [44]	2024	TripAdvisor reviews; Gandhi vs. Pilar de la Horadada (pre-tested).	2 × 2 × 2 (Study 1)/mixed design (Study 2); randomized scenarios.	Outlier data identified using a mean ± 2 standard deviation threshold.	Fixation duration (+), Fixation count (+), Revisit count (+)
Calderón-Fajardo et al. [21]	2024	15 city logos + 5 AI-generated images (DALL-E).	3 phases: individual, comparison, and AI imagery; random order.	Non-parametric tests (Mann–Whitney and Wilcoxon) chosen due to the non-normal distribution of subjective responses.	Time to first fixation (+), Fixation count (+), Fixation duration (+), GSR (+)
Li et al. [45]	2024	36 formal ICH images (Ctrip); expert validated; brightness, contrast and size normalized.	Single-factor experimental; unlimited browsing; FAVORITE project click.	Retrospective oral report to confirm results	Fixation counts (+), Fixation duration (+)
Wasaya et al. [46]	2024	13 pictures of Australian heritage (RealEye platform).	Quantitative survey + 60 s eye-tracking experiment; random image order.	Harman’s single factor test applied to check for common method bias; multicollinearity diagnosed using the Variance Inflation Factor; data normality confirmed	Fixation duration (+)
Ye et al. [47]	2024	60 photos of Minhe Village (Ming/Qing style); brightness normalized.	3 phases (entry, core, departure); 10 s display; 3 s black screen interval.	Outliers removed; pupil diameter measurements normalized using a baseline period before each stimulus.	Fixation duration (TDF) (+), Fixation count (+), Saccades (+), Pupil size (+)
Chang et al. [48]	2025a	36 processed images (Ctrip); Photoshop controlled targets.	Mixed design; random group viewing and randomized trials; keyboard-controlled viewing time.	Only data samples achieving over a 60% sampling rate retained; calibration was accepted only if the bidirectional offset was less than 0.5°.	Fixation duration (+), First fixation duration (+)
Chang et al. [49]	2025b	On-site videos (Hui Min St) vs. social media images.	Free-viewing eye-tracking + physiological (EDA/EMG) sensors.	Physiological artefacts from movement or perspiration identified and removed.	Fixation duration (+), Fixation count (+), Time to first fixation (+)
García-Carrión et al. [50]	2025	8 Facebook posts (Buyuada fictitious destination); pre-tested.	2 × 2 within-subject; tasks performed inside fMRI scanner; fixed display times.	A four-stage ocular cleaning procedure refined data using duration and distance thresholds; pupil area measurements normalized.	Saccadic duration (+), Pupil size (+), Fixation duration (+), Fixation count (+)
Jin et al. [51]	2025	18 real pictures (DMO/UGC); expert reviewed.	2 × 3 within-subject; 8 s display; randomized picture order.	Participants excluded if they showed insufficient attention to the screen; fixed viewing time to reduce individual processing speed differences. Eye-tracking metrics analyzed using R.	Fixation duration (+), Fixation counts (+)
Jóźwiak [52]	2025	3 tourism offer layouts (A, B, C); varied sustainability content.	Individual completion; 55 s display per offer (random order); post-test survey.	Scanpaths and heatmaps cross-validated survey responses.	No sig. effects.
Li et al. [53]	2025	2 opera video clips (Trial in Three-Judge Court); expert selected.	Mixed-method; simulated opera theatre; 7 min video viewing.	Outliers identified and eliminated based on specific z-value thresholds. Confirmatory Tetrad Analysis (CTA) was conducted to determine the suitability of the measurement model.	Fixation duration (+), Emotional peaks (EDA) (+)
Sushchenko et al. [54]	2025	Custom tourism website (tourism-ua.space).	5-block stimuli division (Welcome, Map, Tours, News, Staff); calibration; limited-time free browsing.	Software to denoise measures and remove artefacts.	No sig. effects.

**Table 5:** A mini-guide to statistical modelling of eye-tracking data.

Outcome Type	Metric Examples	Common Error	Recommended Distribution
Count Data (*Discrete, Non-negative*)	• Fixation Count • Revisit Count • Blink Count	Using ANOVA/Linear Regression (assumes normality & continuous data) [33,39,44].	Poisson (if mean ≈ \approx ≈ variance) OR Negative Binomial (if variance > mean, i.e., overdispersed).
Continuous Duration (*Positively Skewed*)	• Fixation Duration • Time to First Fixation • Total Dwell Time	ANOVA on raw data without transformation [35,38,41].	Gamma (with log link) OR Log-Normal (log-transform data, then use Gaussian).
Binary/Probability (*Yes*/*No*)	• AOI Hit (Looked vs. Not looked) • Skip Rate	Chi-square (ignores subject variability) [40,50].	Binomial (logit link).

## Data Availability

Data available upon request.

## Supplementary Materials

The following supporting information can be downloaded at https://www.mdpi.com/article/10.3390/jemr19020031/s1, Table S1: Deduplication Log (with example articles); Table S2: Appraisal Codebook.

## Appendix A

**Table A1:** Checklist for Eye-Tracking Rigour (CETR).

Section	Item#	Checklist Items	Yes	Page#	Recommendation
**1. Design & Theory**	1.1	Is the study grounded in a cognitive framework?	□		Move beyond macro-theories (e.g., S-O-R); integrate cognitive theories (e.g., Attention Resource Theory).
	1.2	Are hypotheses directional and specific?	□		Specify *which* eye movement metric is expected to change and *why*.
	1.3	Is the apparatus appropriate for the task?	□		Use >250 Hz desktop trackers for text-based tasks; use mobile glasses for real-world interaction; use standard screen-based for scene viewing.
**2. Stimulus Control**	2.1	Are visual properties controlled?	□		Report luminance, contrast, and size of stimuli. If comparing images, normalize them or include them as random effects in the model.
	2.2	Are Areas of Interest (AOIs) standardized?	□		Ensure AOIs are of comparable size across conditions or normalize metrics by AOI area/size.
	2.3	Is the trade-off between precision and ecological validity justified?	□		For micro-tasks, prioritize precision (chin rest) and acknowledge artificiality. For macro/exploration tasks, prioritize ecological validity (mobile/remote) and acknowledge reduced accuracy.
**3. Data Acquisition**	3.1	Is calibration reported?	□		Report the calibration method (e.g., 9-point), the average spatial accuracy (in degrees) and calibration intervals.
	3.2	Is the sampling rate sufficient?	□		Ensure the sampling rate can serve the metric purpose (e.g., >250 Hz for microsaccades, >60 Hz for fixations).
	3.3	Is the lighting environment constant?	□		Crucial for pupillometry. Report lux levels at the eye and screen.
**4. Data Processing**	4.1	Were fixations defined?	□		Report the algorithm (e.g., I-VT, I-DT) or data handling procedure that defines fixation thresholds (e.g., velocity > 30°/s, duration > 80 ms).
	4.2	Was data quality reported?	□		Report the percentage of data loss (track loss) and the criteria for excluding participants (e.g., >20% loss).
	4.3	Were outliers removed?	□		Specify the criteria for outlier removal (e.g., fixations < 60 ms or >1000 ms) and the percentage of data removed.
**5. Statistical Analysis**	5.1	Is the model appropriate for the data distribution?	□		Use GLMMs (Poisson/Negative Binomial) for count data; use Gamma/Log-Normal for duration data. Avoid ANOVA on raw count data.
	5.2	Are random effects included?	□		Participant and stimuli differences cannot be fully controlled. Include (1|Subject) and (1|Stimulus) in the analyses.
	5.3	Are data triangulated?	□		Use multiple eye-tracking metrics; triangulate eye-tracking data with self-reports, interviews, or other physiological measures to confirm cognitive interpretation.
