# Supplementary material for: Towards Rigorous Eye-Tracking Methodology in Interdisciplinary Fields: Insights from and Recommendations for Tourism Research
Source: J Eye Mov Res. 2026 Mar 12;19(2):31. doi: 10.3390/jemr19020031 (PMC13010711; doi:10.3390/jemr19020031)
Supplement: Supplementary file 1 [file jemr-19-00031-s001.zip › jemr-4103589-supplementary.pdf]

Supplementary Materials

Table S1. Deduplication Log (with example articles)

Records	WoS	Scopus	Remarks
Deng, W., Lin, Y., & Chen, L. (2021). Exploring destination choice intention by using the tourism photographic: From the perspectives of visual esthetic processing. *Frontiers in Psychology, 12*, 713739. https://doi.org/10.3389/fpsyg.2021.713739	1	1	Duplicate
…			
Sushchenko, O., Kasenkova, K., Pohuda, N., & Petrova, M. (2025). Implementation of Eye-Tracking Technology in the Domestic Tourism Marketing Complex. *Tourism and Hospitality, 6*(2), 94. https://doi.org/10.3390/tourhosp6020094	0	1	Unique
…			
Total records processed	81	149	230 raw record counts
Similar records	81	81	WoS 100% overlaps with Scopus
Unique records	0	68	
Records to enter screening	0	149	WoS duplicates removed

Table S2: Appraisal Codebook

Overview: This codebook outlines the criteria used to evaluate the 23 included studies across eight methodological dimensions. Each dimension was coded based on the presence (1) or absence (0) of specific reporting standards or methodological choices, or categorized nominally where appropriate.

1. Research Design Appropriateness
Evaluates the suitability of the experimental design for the stated research questions.

a. **Design Type:** (Nominal)
    - *Between-subjects:* Participants view only one condition.
    - *Within-subjects:* Participants view all conditions.
    - *Mixed:* Combination of between and within factors.

b. **Control Conditions:** (Binary)
    - *1 (Present):* Study includes a control group or control condition (e.g., viewing a non-tourism image).
    - *0 (Absent):* Study lacks a baseline or control comparison.

c. **Sample Size Justification:** (Binary)

- *1 (Reported):* A priori power analysis or reference to standard field norms provided.
- *0 (Not Reported):* No justification for *N* provided.

2. Apparatus Selection & Technical Decisions

Evaluates the hardware and software specifications relative to the task.

a. **Tracker Type:** (Nominal)
   - *Screen-based (Remote):* e.g., Tobii Pro Spectrum, EyeLink 1000.
   - *Wearable (Glasses):* e.g., Tobii Pro Glasses 2.
   - *Webcam-based:* e.g., RealEye.

b. **Sampling Rate:** (Continuous/Ordinal)
   - Reported in Hz (e.g., 60Hz, 120Hz, 300Hz, 1000Hz).
   - *Code 0 if not reported.*

c. **Accuracy/Precision Reporting:** (Binary)
   - *1 (Reported):* Spatial accuracy (e.g., <0.5°) or precision (RMS noise) explicitly stated.
   - *0 (Not Reported):* No technical error metrics provided.

3. Theoretical Foundation

Evaluates the depth of theoretical integration.

a. **Theoretical Framework:** (Nominal)
   - *Social Science/Marketing:* e.g., S-O-R, Push-Pull, Destination Image.
   - *Cognitive/Psychological:* e.g., Eye-Mind Hypothesis, Spotlight Theory, Limited Capacity Model.
   - *None:* Atheoretical/Purely exploratory.

b. **Hypothesis Type:** (Binary)
   - *1 (Confirmatory):* Specific directional hypotheses regarding eye movements (e.g., "H1: Higher complexity will increase fixation duration").
   - *0 (Exploratory):* General research questions without specific predictions (e.g., "To explore where tourists look").

4. Stimulus Design & Validation

Evaluates the quality and control of the visual materials.

a. **Stimulus Modality:** (Nominal)
   - *Static:* Images, text, screenshots.
   - *Dynamic:* Video, VR, scrolling websites.

b. **Low-Level Visual Control:** (Binary)
   - *1 (Controlled):* Stimuli matched for luminance, contrast, size, or complexity (e.g., using histograms or saliency maps).
   - *0 (Uncontrolled):* Raw images used without modification or measurement of visual properties.

c. **Stimulus Validation:** (Binary)
   - *1 (Validated):* Stimuli pre-tested or rated by experts/participants for content validity (e.g., "perceived luxury").
   - *0 (Not Validated):* Stimuli selected by authors without external validation.

5. Data Collection Procedures

Evaluates the rigor of the experimental protocol.

a. **Calibration Procedure:** (Binary)
   - *1 (Reported):* Details provided (e.g., 5-point or 9-point calibration, validation check before recording).
   - *0 (Not Reported):* No mention of calibration steps.

b. **Viewing Conditions:** (Binary)
   - *1 (Controlled):* Viewing distance, lighting conditions, or chin-rest usage reported.
   - *0 (Unspecified):* Environmental conditions not described.

c. **Task Instruction:** (Nominal)
   - *Free-viewing:* "Look at these images."
   - *Goal-directed:* "Find the price," "Rate the likelihood to visit."

6. Data Quality & Cleaning

Evaluates how raw data was processed before analysis.

a. **Data Loss Reporting:** (Binary)
   - *1 (Reported):* Percentage of trials or participants excluded due to poor tracking quality reported.
   - *0 (Not Reported):* No mention of data exclusion rates.

b. **Fixation Identification Algorithm:** (Binary)
   - *1 (Defined):* Algorithm (e.g., I-VT, I-DT) or thresholds (e.g., dispersion >100px, duration >80ms) specified.
   - *0 (Undefined):* No information provided.

c. **Outlier Handling:** (Binary)
   - *1 (Systematic):* Criteria for removing outliers (e.g., +/- 3 SD) defined.
   - *0 (Ad-hoc/None):* No outlier removal mentioned.

7. Statistical Analysis Appropriateness

Evaluates the statistical models used for eye-tracking data.

a. **Model Type:** (Nominal)
   - *Basic Parametric:* t-test, ANOVA (F-test).
   - *Advanced/Mixed:* Linear Mixed Models (LMM), Generalized Estimating Equations (GEE).
   - *Non-parametric:* Mann-Whitney U, Kruskal-Wallis.

b. **Correction for Multiple Comparisons:** (Binary)
   - *1 (Applied):* Bonferroni, Holm, or similar corrections used for multiple AOIs or metrics.
   - *0 (Not Applied):* Uncorrected p-values reported.

8. Reporting Completeness

Evaluates the overall transparency of the manuscript.

a. **Metric Definition:** (Binary)
   - *1 (Explicit):* Metrics clearly defined (e.g., "Total Fixation Duration = sum of all fixations within AOI").
   - *0 (Implicit):* Metrics used without definition.

b. **Visualizations:** (Binary)
   - *1 (Descriptive Only):* Heatmaps/Gaze plots used only for illustration.
   - *0 (Inference by Eye):* Conclusions drawn directly from visual inspection of heatmaps without statistical backing.
